# Short-term effects of CO_2_ leakage on the soil bacterial community in a simulated gas leakage scenario

**DOI:** 10.7717/peerj.4024

**Published:** 2017-11-14

**Authors:** Jing Ma, Wangyuan Zhang, Shaoliang Zhang, Qianlin Zhu, Qiyan Feng, Fu Chen

**Affiliations:** 1 Low Carbon Energy Institute, China University of Mining and Technology, Xuzhou, China; 2 School of Environment Science and Spatial Informatics, China University of Mining and Technology, Xuzhou, China

**Keywords:** CO_2_ geological storage, Potential CO_2_ leakage, Acidobacteria, Methylophilus, Soil enzyme activity

## Abstract

The technology of carbon dioxide (CO_2_) capture and storage (CCS) has provided a new option for mitigating global anthropogenic emissions with unique advantages. However, the potential risk of gas leakage from CO_2_ sequestration and utilization processes has attracted considerable attention. Moreover, leakage might threaten soil ecosystems and thus cannot be ignored. In this study, a simulation experiment of leakage from CO_2_ geological storage was designed to investigate the short-term effects of different CO_2_ leakage concentration (from 400 g m^−2^ day^−1^ to 2,000 g m^−2^ day^−1^) on soil bacterial communities. A shunt device and adjustable flow meter were used to control the amount of CO_2_ injected into the soil. Comparisons were made between soil physicochemical properties, soil enzyme activities, and microbial community diversity before and after injecting different CO_2_ concentrations. Increasing CO_2_ concentration decreased the soil pH, and the largest variation ranged from 8.15 to 7.29 (*p* < 0.05). Nitrate nitrogen content varied from 1.01 to 4.03 mg/Kg, while Olsen-phosphorus and total phosphorus demonstrated less regular downtrends. The fluorescein diacetate (FDA) hydrolytic enzyme activity was inhibited by the increasing CO_2_ flux, with the average content varying from 22.69 to 11.25 mg/(Kg h) (*p* < 0.05). However, the increasing activity amplitude of the polyphenol oxidase enzyme approached 230%, while the urease activity presented a similar rising trend. Alpha diversity results showed that the Shannon index decreased from 7.66 ± 0.13 to 5.23 ± 0.35 as the soil CO_2_ concentration increased. The dominant phylum in the soil samples was *Proteobacteria*, whose proportion rose rapidly from 28.85% to 67.93%. In addition, the proportion of *Acidobacteria* decreased from 19.64% to 9.29% (*p* < 0.01). Moreover, the abundances of genera *Methylophilus*, *Methylobacillus*, and *Methylovorus* increased, while *GP4*, *GP6* and *GP7* decreased. Canonical correlation analysis results suggested that there was a correlation between the abundance variation of *Proteobacteria*, *Acidobacteria*, and the increasing nitrate nitrogen, urease and polyphenol oxidase enzyme activities, as well as the decreasing FDA hydrolytic enzyme activity, Olsen-phosphorus and total phosphorus contents. These results might be useful for evaluating the risk of potential CO_2_ leakages on soil ecosystems.

## Introduction

Under the background of the rapid development of the world economy and the consumption of fossil fuels, global warming has become one of the most urgent challenges for human development and survival. Carbon dioxide (CO_2_) capture and storage (CCS) technology was developed to separate and collect the CO_2_ from the point source and inject to deep reservoir or salt water layer for long-term sequestration (Zhou et al., 2013; Ko et al., 2016; Li et al., 2016). The CCS method has a huge potential for long-term CO_2_ emission reduction, and also has the advantage of low cost. Although CCS technology has made a great contribution to alleviating the global greenhouse effect, there remains a risk of leakage from geological storage. A strong, rapid leakage of CO_2_ gas could cause huge losses of life and property. Studies have shown that once CO_2_ leakage occurred, the concentration of CO_2_ in the air may increase to 3–5%. As CO_2_ is an asphyxiant gas, this concentration could cause headache, dizziness, nausea, and even death following long-term exposure (West et al., 2005; Patil, Colls & Steven, 2010; Tian et al., 2013; Andrzej & Katarzyna, 2013). Furthermore, slow leakage of CO_2_ into soil can lead to variations of soil gas composition, moisture, pH, and subsequently, microbial communities (Beaubien et al., 2008). This would eventually result in the change of the soil ecological environment. Therefore, it is crucial to be prepared to deal with the possibility and evaluate its risk on the soil environment (Sáenz de Miera et al., 2016). In addition, information regarding the impacts of accidental leakage on near surface soil communities has gradually enriched. Considering that bacteria are ubiquitous and possess tremendous metabolic and physiological versatility, they are an essential part in the soil ecosystem and are critical to virtually all biogeochemical processes (Horner-Devine, Carney & Bohannan, 2004). Moreover, bacteria are the most abundant and diverse group of soil microorganisms (Acosta-Martínez et al., 2010). Therefore, it is especially important to understand how the microbial community responds to CO_2_ gas leakage (Prosser, 2007; Acosta-Martínez et al., 2008; Van Der Heijden, Bardgett & Van Straalen, 2008; Sáenz de Miera et al., 2014).

In recent years, the rapid development of culture-independent methods has gradually led to increased research focus on microbial community diversity and composition. This has provided new horizons for assessing the reactions of bacterial community to external disturbances, such as elevated CO_2_ concentration (Dumbrell et al., 2010; Lemos et al., 2011; Maček et al., 2011; Mandić-Mulec et al., 2012; Sáenz de Miera et al., 2016; Schloss et al., 2009; Tait et al., 2015; Taylor et al., 2014). The increasing CO_2_ concentration could bring about a variation of soil biochemical conditions, which would cause the shifting diversity or functionality of indigenous bacteria (Fernández-Montiel et al., 2015). However, the elevated CO_2_ could directly affect species richness, or it may trigger changes in the composition without any discernible effect on diversity (Morales & Holben, 2014). The degradation of organic matter, as well as the biogeochemical cycling of nutrients may also be impacted (Tait et al., 2015).

Many researchers have investigated the impacts of CO_2_ leakages from geological sources in natural sites (Frerichs et al., 2013; Gabilondo & Bécares, 2014; Krüger et al., 2011; McFarland, Waldrop & Haw, 2013; Oppermann et al., 2010; Sáenz de Miera et al., 2014). Considering the long periods of CO_2_ emissions from natural seepages, the ecosystem might have already adapted through species substitution (Krüger et al., 2011). Furthermore, these natural sites have presented existing special features, such as soil type, soil moisture and temperature, which might have not appeared in the potential scenario of a CCS leakage. Therefore, the results from the natural sites may not be extrapolated to the conditions occurring in other areas after possible leakage from the CCS process (Ziogou et al., 2013; Fernández-Montiel et al., 2015). Therefore, the current study was carried out in an experimental site in an attempt to assess the short-term effects on bacterial communities rather than the long-term effects. The aim was to analyze the first responses of a non-adapted ecosystem to simulated CO_2_ leakage. Also, the consequences of the underground low gas emissions through the quantitative evaluation of soil microbial communities were investigated. The impacts on the soil bacterial community from the simulated below-ground CO_2_ emissions were examined. In addition, a limited understanding of the importance of abiotic factors in regulating biodiversity and the structure of some important soil bacterial communities were also investigated (West et al., 2009; Chen et al., 2017). Moreover, soil enzymes are essential to the soil ecosystem for the material cycle and energy conversion. All biochemical reactions in the soil are catalyzed by soil enzymes, which also reflects the strength and direction of the biochemical processes in soil (Sardans, Penuelas & Estiarte, 2008; Duan et al., 2015). Therefore, the soil enzyme activity can be used as an important indicator to evaluate the soil fertility. Usually many factors can affect soil enzyme activities, such as soil physicochemical properties, soil biota, agricultural vegetation and the human factor (Naidu & Sarathambal, 2012; Sarathambal et al., 2016). However, few studies have examined the effect of high soil CO_2_ levels on microbial enzyme activities (Beulig et al., 2016).

In this study, a small simulation experiment, which includes an automatic control auxiliary device and continuous CO_2_ injection, was established to simulate the leakage from a geological storage site. This simple simulation device was cheap and practical, fully meeting the demand for investigating the short-term effects on the bacterial community, which was different from our previous field simulation experimental platform (Chen et al., 2016). The relationship between environmental factors and the soil bacterial community was also studied. The ecosystem functions could be changed overall by the community composition changes that enhance the presence of several taxa. Such knowledge will be useful to understand the potential effects of CO_2_ leakages from geological storage sites on soil ecosystems in the future.

## Materials and Methods

### Study site and experimental design

**Figure 1:**
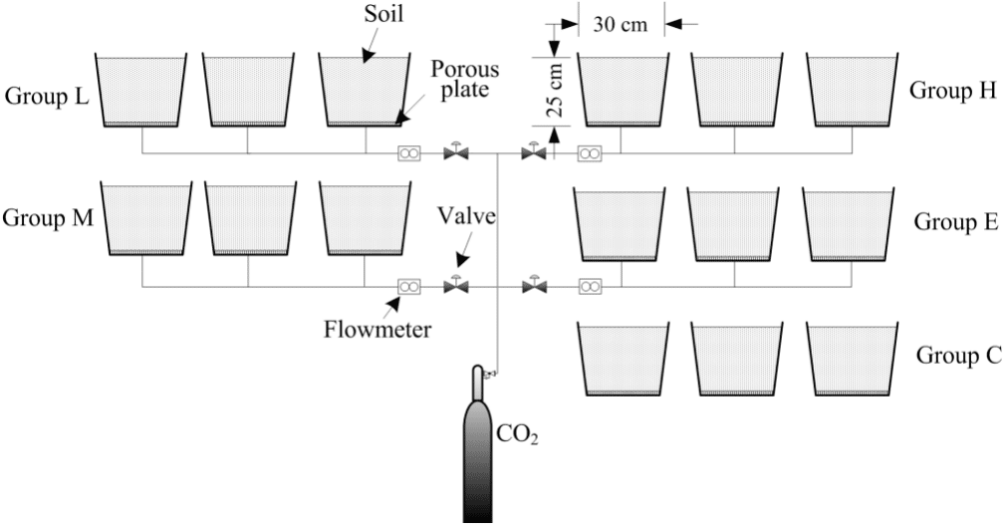
Schematic of the CO_2_ leakage simulation experimental platform.

This experiment was established to simulate CO_2_ leakage during CCS. This study was conducted at the Wenchang campus of China University of Mining and Technology (117.206194°E, 34.229568°N), Xuzhou, Jiangsu Province, China. The experiment contained five treatments, each with three replicates. The treatments consist of the following CO_2_ gas flux levels: low (L, 400 g  m^−2^ day^−1^), medium (M, 1,000 g m^−2^ day^−1^), high (H, 1,500 g m^−2^ day^−1^), extreme (E, 2,000 g m^−2^ day^−1^) and a control with no CO_2_ injection (C). Containers used in this experiment were 30 cm (height) × 30 cm (diameter) and filled with a 25 cm topsoil layer. A layer of porous plate was placed at a distance of 5 cm from the container bottom. A CO_2_ output control device was formed by connecting the cylinders, pressure reducing valve, diverter, PVC hose and other devices. The PVC hose was inserted into the center of the bottom of the container and fixed at the 2–3 cm mark in the bottom section above the outlet position (Fig. 1). During the experiment, the 5-cm space was filled with CO_2_ gas, and the gas spread evenly into the soil.

By adjusting the pressing deducing valve and the adjustable flow meter, the uninterrupted CO_2_ gas leaking into the soil in the four treatments were maintained treatment levels. The experiment began on March 19, 2016 (T0, before ventilation) and several samplings days took place subsequently (April 1st, April 16th, May 1st, June 3rd (denoted as T1, T2, T3, and T4, respectively). The ventilation was then stopped and final sampling took place on July 4th (T5) to investigate the restoration of the soil ecological environment after the CO_2_ stress was relieved.

### Sample collection and soil property analysis

Approximately 100 g soil samples were collected from five points from the middle position of each container at about 10 cm deep and then were mixed to bulk samples from each container. Next, these mixed samples were put into a sterile sealing bag, and immediately sent to the laboratory. About 5 g soil was stored at −20°C for subsequent analysis of microbial diversity. Other parts were placed directly in indoor shaded conditions for drying. Sand grinding was manually removed through a 2-mm screen. These samples were then passed through the 0.149-mm sieve for the testing of physical and chemical properties.

Soil pH and electrical conductivity (EC) values were measured using a pH meter and conductivity meter, respectively (PHC-3C, DDS-307A, Shanghai Leici, China). Organic matter (OM) content was measured with colorimetric methods using hydration heat during the oxidation of potassium dichromate (Chen et al., 2016). Total nitrogen content was measured by dual wavelength spectrometry (Norman, Edberg & Stucki, 1985). The total phosphorus content was determined by the acid digestion molybdenum and antimony colorimetric method. The available phosphorus was measured by the hydrochloric acid ammonium chloride method.

Soil urease activity was measured by the phenol colorimetry of sodium hypochlorite (Guan, 1986). Polyphenol oxidase activity was determined by pyrogallol colorimetry (Guan, 1986). Protease activity was measured by the ninhydrin colorimetric method (Guan, 1986). The triphenyltetrazolium chloride (TTC) method was used to measure soil dehydrogenase activity (Guan, 1986). The fluorescein diacetate (FDA) hydrolysis activities were determined by the fluorescein colorimetric method (Guan, 1986). Three repetition groups were set up in each test. The negative control groups were provided that had either no substrate or no soil.

### DNA extraction, PCR amplification and Illumina MiSeq sequencing

According to the instruction manual, 20 soil samples of DNA were extracted from 0.5 g fresh soil samples with the E.Z.N.A™ Mag-Bind Soil DNA Kit (Omega Biotek, Guangzhou, China). The 20 soil samples were marked as C0–C3, L0–L3, M0–M3, H0–H3, E0–E3 (collected at T0, T1, T2 and T3, respectively). The V3–V4 region of the bacterial 16S rRNA genes were amplified using the primer set 341F (CCCTACACGACGCTCTTCC GATCTG-CCTACGGGNGGCWGCAG) and 805R (GACTGGAGTTCCTTGGCACCCGA GAATTCCA-GACTACHVGGGTATCTAATCC). The 50 µL PCR reaction system included 5 µL of 10 × PCR buffer, 0.5 µL of each primer (50 µM), 0.5 µL of dNTP (10 mM each), 0.5 µL Platium Taq (5U/uL), 42 µL sterilized ultrapure water and 1 µL of template DNA (10 ng/ µL). Amplification conditions were as follows: 3 min at 94°C, 5 cycles of 94°C for 30 s, 45°C for 20 s, and 65°C for 30 s, 20 cycles of 94°C for 20 s, 55°C for 20 s, and 72°C for 30 s and final 5 min incubation at 72°C to complete the extension process. According to the instruction manual, PCR products were pooled and purified with the Agencourt AMPure XP (Beckman, Indianapolis, IN, USA). Quantification of the purified PCR products was carried out using the Qubit2.0 test kit (Life Technologies, Carlsbad, CA, USA). Finally, 20 pmol DNA was analyzed sequentially using the Illumina MiSeq platform (Sangon Biotech, Shanghai, China).

### Bioinformatics analysis

The sample sequencing data were distinguished with the barcode sequence, and the sequence of each sample underwent quality control. Then the non-specific amplification sequences and chimeric were removed with the usearch method (http://www.drive5.com/usearch/). Operational taxonomic units (OTUs) with a 97% similarity cutoff were also clustered using the usearch method. Mothur software was used to analyze the alpha diversity (http://www.mothur.org/). The Chao (http://www.mothur.org/wiki/Chao) and Shannon indices (http://www.mothur.org/wiki/Shannon) were conducted to reveal the richness and diversity. A RDP classifier was used to classify the species (http://rdp.cme.msu.edu/misc/resources.jsp). According to the taxonomic results, a species’ abundance diagram and rich infrared images were constructed with Origin8 and R software. The beta diversity analysis was performed using the weighted UniFrac metric algorithmic with R software. Canonical correlation analysis (CCA) was used to study the relationship between environmental stressors and microbial communities with Canoco 4.5 for windows software. SPSS 20 software was used to analyze the data of soil properties and enzyme activities and spearman correlations were calculated. Significance for all statistical analyses was accepted at *α* = 0.01 or *α* = 0.05. One-way ANOVA was performed and Duncan’s test were applied in the case of multiple comparisons.

## Results

### Effects of CO_2_ leakage on the soil characteristics

**Figure 2:**
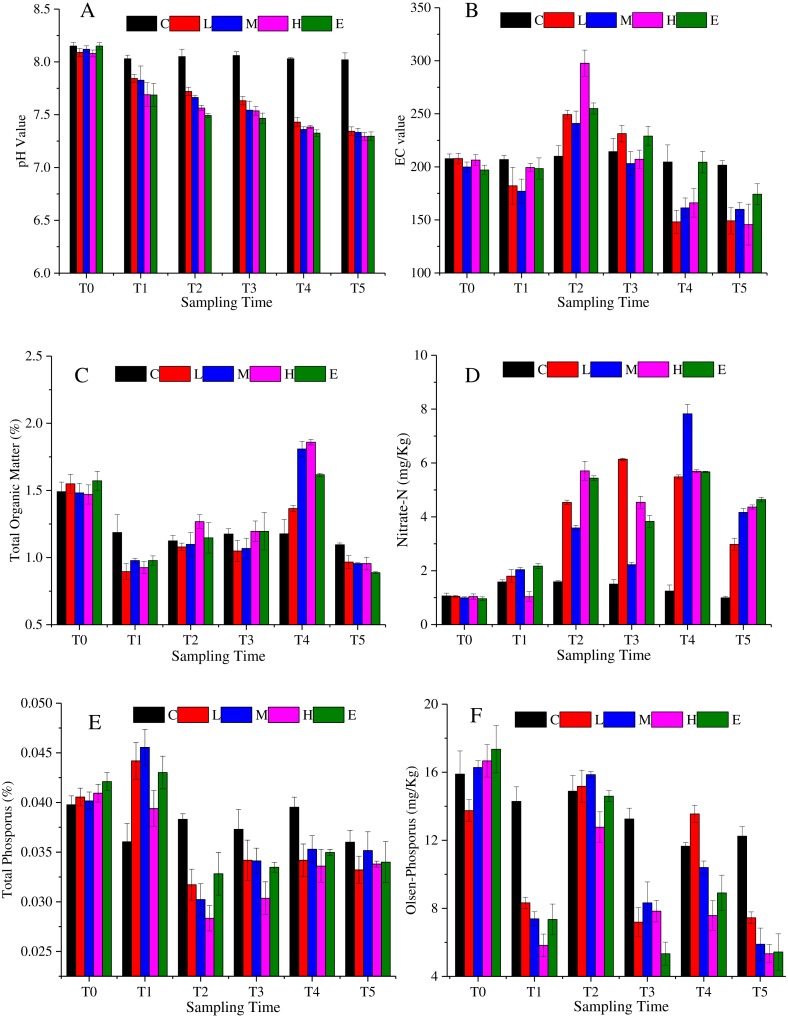
Soil physicochemistry change under different CO_2_ flux, (A) pH value; (B) EC value; (C) total organic matter; (D) nitrate-N; (E) total phosphorus; (F) Olsen-phosphorus. (C, Control; L, 400 g m^−2^ d^−1^; M, 1,000 g m^−2^ d^−1^; H, 1,500 g m ^−2^ d^−1^; E, 2,000 g m^−2^ d^−1^).

The soil characteristics are summarized in Fig. 2. Figure 2A shows that soil pH changed significantly under CO_2_ stress (*p* < 0.05). The pH value of C treatment showed a slight fluctuation. The value was 8.15 at T0, then it basically maintained at about 8.04 from T1 to T5. Considering the environmental impact of the test itself, the pH value of C treatment can be considered stable. The L, M, H and E treatments were significantly different from the C treatment (*p* < 0.05). The pH decreased significantly with the increasing CO_2_ concentration. The spearman correlation coefficient was between −0.9 and −1 from T1 to T5. Eventually, at T5, the soil pH of the L and M treatments dropped to about 7.34, whereas the soil pH under the H and E treatments reached about 7.29. Figure 2B demonstrates that the EC first increased and then decreased overtime. The maximum EC value was detected at T2. Furthermore, the H treatment showed the maximum variation amplitude. Also, similar values at T4 and T5 in C and E treatments were observed. The soil organic matter showed a tendency to decrease abruptly and then increase (Fig. 2C). During the period of T0–T1, the content of organic matter decreased substantially, and then increased from T1 to T4. Once again, the organic matter content decreased in the recovery period (T5), and the value became lower than the initial content. Overall, the rates of change were greater in the L, M, H, and E treatments than that in the C treatment.

The nitrate nitrogen content of all treatments increased over time with some fluctuations (Fig. 2D). The average value of the L, M, H, and E treatments was about 1.01 mg/Kg at T0, whereas the value reached 4.03 mg/Kg at T5. The nitrate nitrogen value of the L, M, H, and E treatments increased significantly from T1 to T2. At T3, nitrate nitrogen values of the M, H and E treatments decreased slightly and then continued rising to T4 (*p* < 0.05). However, in the recovery period, the value declined again. The change trend of the total phosphorus was shown in Fig. 2E, which accumulated at T1, and then decreased at T2. In other words, after half a month of ventilation, the basic data increased and the increase amplitude was between 2.5% and 10% (T1). The value then suddenly fell approximately 25% ∼ 33% at T2 (Fig. 2E). Also, from Fig. 2E it can be speculated that there was a tendency to decrease at T2–T5 when CO_2_ is applied. Furthermore, the overall trend of the soil Olsen-phosphorus was complex (Fig. 2F). The Olsen-phosphorus in the C treatment fluctuated, but the L, M, H, and E treatments also fluctuated but at a significantly higher concentration. For example, the results of L treatment from T0 to T5 were 13.75 mg/Kg, 8.32 mg/Kg, 15.18 mg/Kg, 7.19 mg/Kg, 13.55 mg/Kg, and 7.44 mg/Kg, respectively. The M, H and E treatments had similar fluctuations to the L treatment presenting a different response.

### Effects of CO_2_ leakage on the soil enzyme activity

The redox enzyme plays an important role in the process of material transformation in soil. The transformation of organic matter, the formation of humus and its components are all related to the oxidation of the soil enzyme, such as dehydrogenase and polyphenol oxidase. Through the analysis of soil enzyme activity, the transformation of some nutrients and the status of soil fertility might be understood and predicted.

**Figure 3:**
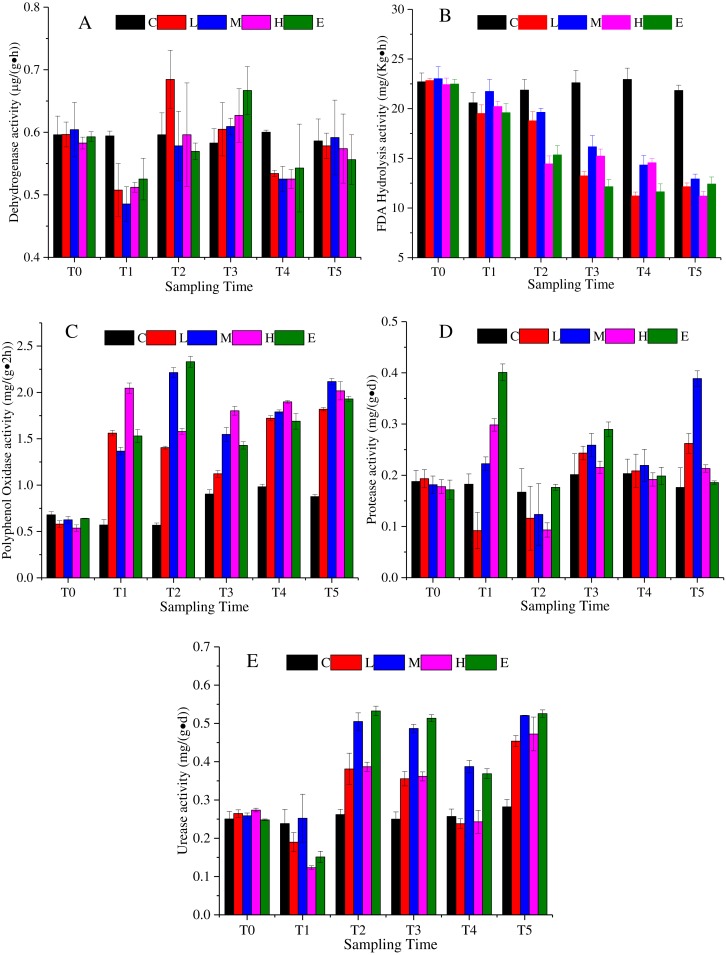
Soil enzyme activity change under different CO_2_ flux, (A) dehydrogenase activity; (B) FDA hydrolysis activity; (C) polyphenol oxidase activity; (D) protease activity; (E) urease activity. (C, Control; L, 400 g m^−2^ d^−1^; M, 1,000 g m^−2^ d^−1^; H, 1,500 g m ^−2^ d^−1^; E, 2,000 g m^−2^ d^−1^).

Dehydrogenase is an enzyme related with respiratory metabolism, and plays the role of hydrogen intermediate transfer body in the dehydrogenation reaction. Figure 3A shows that the dehydrogenase change under the L, M, H, and E treatments appeared as a decrease—rise—decrease tendency with different response times. At T1, excluding the C treatment, dehydrogenase activity of all treatments decreased, and then increased. The rising state of the L treatment ended after T2, while the rising phenomenon of the dehydrogenase activity of M, H, and E treatments lasted until T3 (Fig. 3A) (*p* < 0.05). At the recovery period, they showed a slight rise once again.

The FDA hydrolytic enzyme is widely used in soil quality assessment. The FDA hydrolysis activity presented a downward trend under the effects of CO_2_ leakage (Fig. 3B) (*p* < 0.05). The average value of the L, M, H, and E treatments was 22.69 mg/ (Kg h) at T0, which then reduced to 11.25 mg/ (Kg h) at T5. The response of the L, M, H, and E treatments was uniform, excluding the M treatment, which appeared slightly larger than the other treatments at each time period (Fig. 3B).

Polyphenol oxidase could promote the oxidation of one or two or three phenols, and could also characterize the activity of some soil microorganisms. The activity of polyphenol oxidase showed an upward trend under CO_2_ stress (Fig. 3C) (*p* < 0.05). The mean value of the L, M, H, and E treatments at T4 increased by 230% compared with the average value at T0. During the ventilation experiment, the activities of polyphenol oxidase showed different increasing trends. Compared with that at T4, there was little change of polyphenol oxidase activity at recovery phase.

The protease activity in the ventilation time showed an irregular variation trend in Fig. 3D (*p* < 0.05). For example, the activity of the L treatment firstly decreased at T1, increased at T2 and T3, and then decreased at T4. The activities of the M, H and E treatments increased at T1, declined at T2, increased at T3, and decreased at T4 (Fig. 3D). In the recovery period, the M treatment appeared to rise sharply, which could not be ruled out by the experimental error.

Soil urease is the key enzyme in the conversion of nitrogen in soil. The enzymatic reaction product of urease is one of the plant nitrogen sources, and its activity can be used to indicate the status of soil nitrogen. Figure 3E indicates that the urease activity also showed a complex change with a decrease - rise - decrease tendency (*p* <0.05). The response time of each of the CO_2_-addition treatments was basically consistent, whereas the response level was different. For times after T2, the urease activities of the M and E treatments were significantly higher than those of the L and H treatments. There was an increase in the recovery period (Fig. 3E).

### Effects of CO_2_ leakage on the taxonomic composition of microbial consortia

The richness and diversity of microbial communities can be reflected by the Alpha diversity analysis (Table 1). After comparing the Chao 1 index of each treatment, it can be found that soil bacterial richness in the L and M treatments decreased with the increasing CO_2_ concentration, while the H and E treatments showed a slight increase. In addition, the Shannon index was used to measure the heterogeneity of the community. The data in Table 1 also showed that Shannon index declined with the rising CO_2_ concentration, which indicated that the microbial community diversity in the soil decreased under CO_2_ stress.

**Table 1:** Alpha diversity indices of soil microorganisms (C, Control; L, 400 g m^−2^ day^−1^; M, 1,000 g m^−2^ day^−1^; H, 1,500 g m^−2^ day^−1^; E, 2,000 g m^−2^ day^−1^, where 0–3 represents samples collected at T0–T3, respectively).

Sample ID	Chao 1 index	Shannon index
C0	15,604.19 ± 1,704.233ef	7.65 ± 0.237d
L0	15,601.76 ± 425.667ef	7.66 ± 0.128d
M0	16,543.62 ± 1,022.004f	7.63 ± 0.264d
H0	17,185.51 ± 677.133f	7.454 ± 0.322d
E0	15,136.54 ± 645.322def	7.44 ± 0.113d
C1	14,815.69 ± 2,714.734def	7.38 ± 0.186d
C2	14,666.78 ± 1,144.127def	7.33 ± 0.070d
C3	14,567.04 ± 1,403.888def	7.30 ± 0.036d
L1	12,457.13 ± 1,062.828ab	7.11 ± 0.457d
L2	12,044.64 ± 1,172.405ab	7.03 ± 0.426d
L3	11,238.16 ± 1,177.577a	6.38 ± 0.293c
M1	11,163.37 ± 1,289.129a	6.38 ± 0.846c
M2	10,900.97 ± 1,346.372a	6.03 ± 0.486bc
M3	10,660.81 ± 1,103.014a	6.09 ± 0.544bc
H1	15,105.19 ± 652.463def	6.20 ± 0.041bc
H2	14,614.52 ± 572.322def	6.15 ± 0.040bc
H3	14,509.60 ± 998.913def	6.13 ± 0.523bc
E1	13,398.76 ± 627.144bcd	5.31 ± 0.464a
E2	14,295.70 ± 917.406cde	5.68 ± 0.301ab
E3	13,238.25 ± 216.176bcd	5.23 ± 0.347a

**Figure 4:**
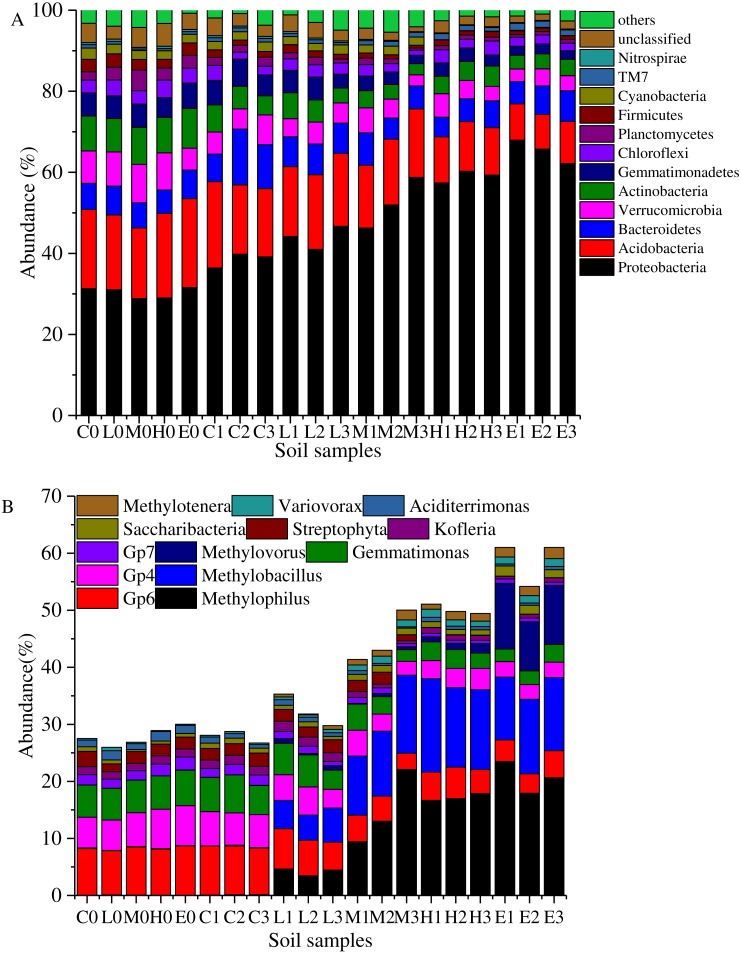
Taxonomic composition of soil samples on Phylum (A) and genus level (B). (C, Control; L, 400 g m^−2^ day^−1^; M, 1,000 g m^−2^ day^−1^; H, 1,500 g m ^−2^ day^−1^; E, 2,000 g m^−2^ day^−1^, where 0–3 represents samples collected at T0–T3, respectively).

Overall, the bacterial categories were relatively abundant in the 20 soil samples (Fig. 4A). *Proteobacteria*, *Acidobacteria*, *Bacteroidetes*, *Verrucomicrobia*, *Actinobacteria*, *Gemmatimonadetes*, *Firmicutes*, *Planctomycetes*, *Cyanobacteria*, *Chloroflexi*, *Candidatus Saccharibacteria* (TM7), and *Nitrospirae* comprised more than 90% of the total sequences in each soil sample. These high proportion phyla (>0.5%) were used to analyze the effects of stress on the soil bacterial structure. The most abundant phylum was *Proteobacteria* which accounted for 28.85%–67.93% (Fig. 4A). Figure 4A shows that the abundant proportion of *Proteobacteria* increased with the increasing CO_2_ concentration. The average proportion was about 30.35% in the C treatment at T0, whereas it increased to 65.28% in the E treatment at T1. The abundance of phylum *Candidatus Saccharibacteria* (TM7) also presented a gradually increasing trend with the rising CO_2_ flux. Furthermore, *Acidobacteria* was the second most abundant phylum. In the C treatment, it demonstrated an average abundance of 19.64% at T0. In the E treatment at T2, it was 9.29%. The higher the CO_2_ stress concentration, the lower the abundance of phylum *Acidobacteria*. Moreover, the abundances of other phyla such as *Verrucomicrobia*, *Actinobacteria*, *Gemmatimonadetes*, *Firmicutes*, *Planctomycetes*, *Cyanobacteria*, *Chloroflexi*, and *Nitrospirae* decreased with the increasing CO_2_ flux (Fig. 4A). Furthermore, the proportion of *Bacteroidetes* showed no significant variability between treatments.

Figure 4B shows the abundance variation at the genus level. The abundances of genera *Methylophilus*, *Methylobacillus* and *Methylovorus* increased rapidly after the ventilation. The average proportions were 0.04%, 0.076% and 0.006% in the C treatment at T0. These numbers increased to 20.68%, 12.30% and 10.16% in the E treatment at T2. The higher the CO_2_ concentration was, the greater was the abundance of genera. In addition, *Saccharibacteria*, *Variovorax* and *Methylotenera* presented the same trend. In contrast, the abundances of *Gp6*, *Gp4*, *Gemmatimonas*, *Gp7* and *Streptophyta* at T0 were 8.23%, 6.12%, 5.81%, 1.87% and 2.05%. These proportions reduced to 3.97%, 2.67%, 2.57%, 0.66% and 0.03% in the E group at T2. *Kofleria* and *Aciditerrimonas* showed the same trend that the higher the concentration of the stress concentration, the smaller the abundance.

### Comparison of bacterial consortia compositions

**Figure 5:**
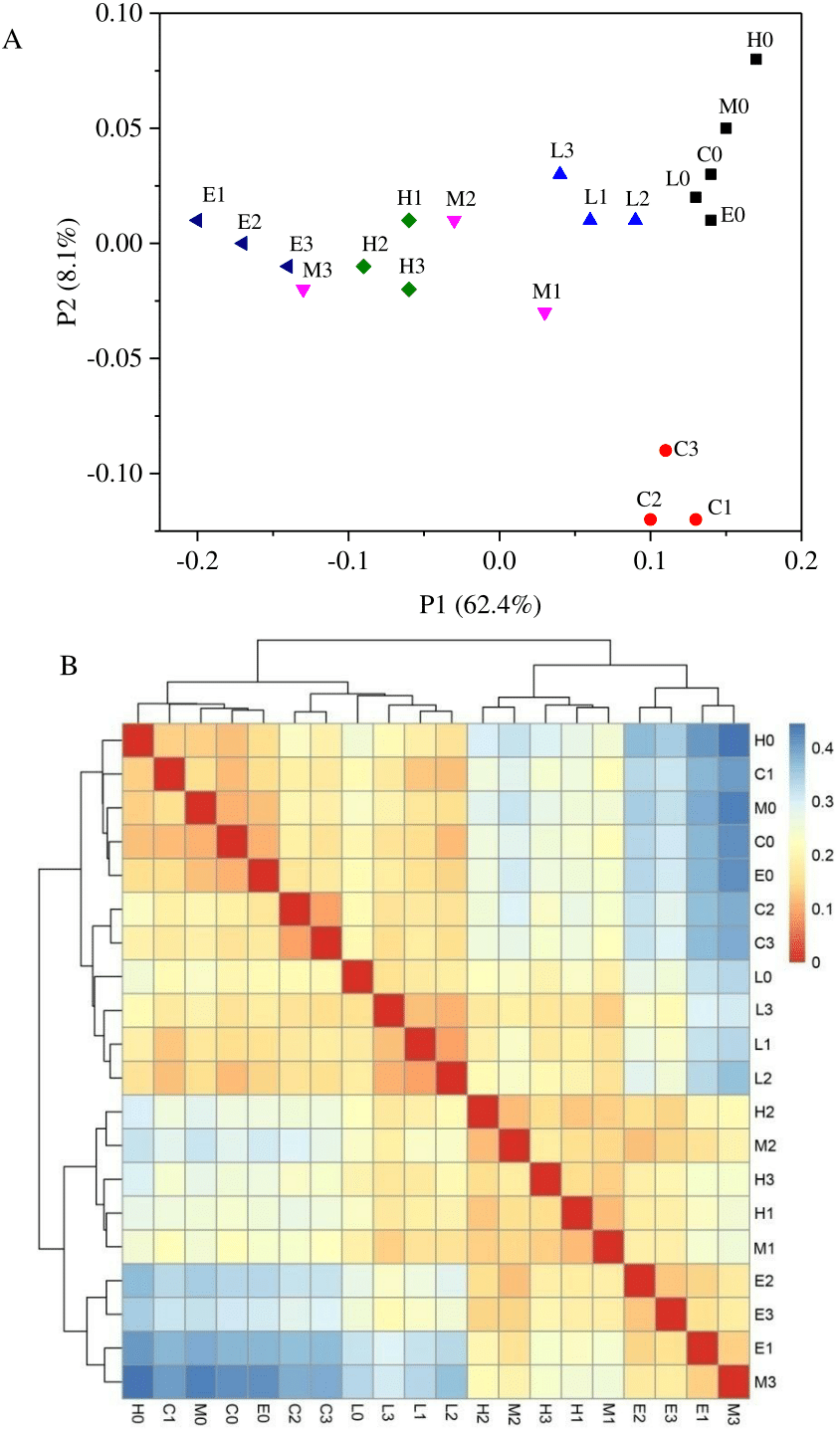
Principal co-ordinates analysis (A) and heatmap of Beta diversity analysis (B) using weighted UniFrac. (C, Control; L, 400 g m^−2^ day^−1^; M, 1,000 g m^−2^ day^−1^, H; 1,500 g m ^−2^ day^−1^; E, 2,000 g m^−2^ day^−1^, where 0–3 represents samples collected at T0–T3, respectively).

Based on the weighted UniFrac algorithms, principal co-ordinates analysis (PcoA) was used to analyze the relation among the 20 samples (Fig. 5A). According to the PcoA, samples with higher similarity gather together, while samples with lower similarity are positioned further apart. Figure 5A shows that every sample of its own group was grouped closely, excluding the samples from the M treatment. At T0, from the C treatment to E treatment, their arrangement presented a pattern along the horizontal axis (Fig. 5A). This analysis also indicated that there were significant differences among these six bacterial community groups with different CO_2_ leakage conditions. A sample distance heatmap was also one of the beta diversity indicators based on the weighted UniFrac algorithms, which presented the distance between the samples. In the heatmap, a color block represents the distance value (Fig. 5B). A deeper red color indicates a higher similarity between the samples, whereas a deeper blue color indicates a greater distance. The samples were also clustered in the heatmap, so the distance between samples can be seen through the cluster tree. As shown in Fig. 5B, the similarity was higher between samples in each group. The C and L treatments had higher similarity, whereas the M, H and E treatments had closer sample distances. Sample distances increased with the increasing CO_2_ concentration, which is supported by the cluster tree.

### Canonical correlation analysis

According to the above experimental results, it can be inferred that soil CO_2_ concentration affected the soil physicochemical properties and microbial community structure, thus affecting the soil enzyme activity. Moreover, the soil community structure could be indirectly affected by the change of physicochemical properties and enzyme activity. Subsequently, CCA was used to analyze the correspondence between the environmental factors and microbial community composition under different CO_2_ ventilation concentration.

**Figure 6:**
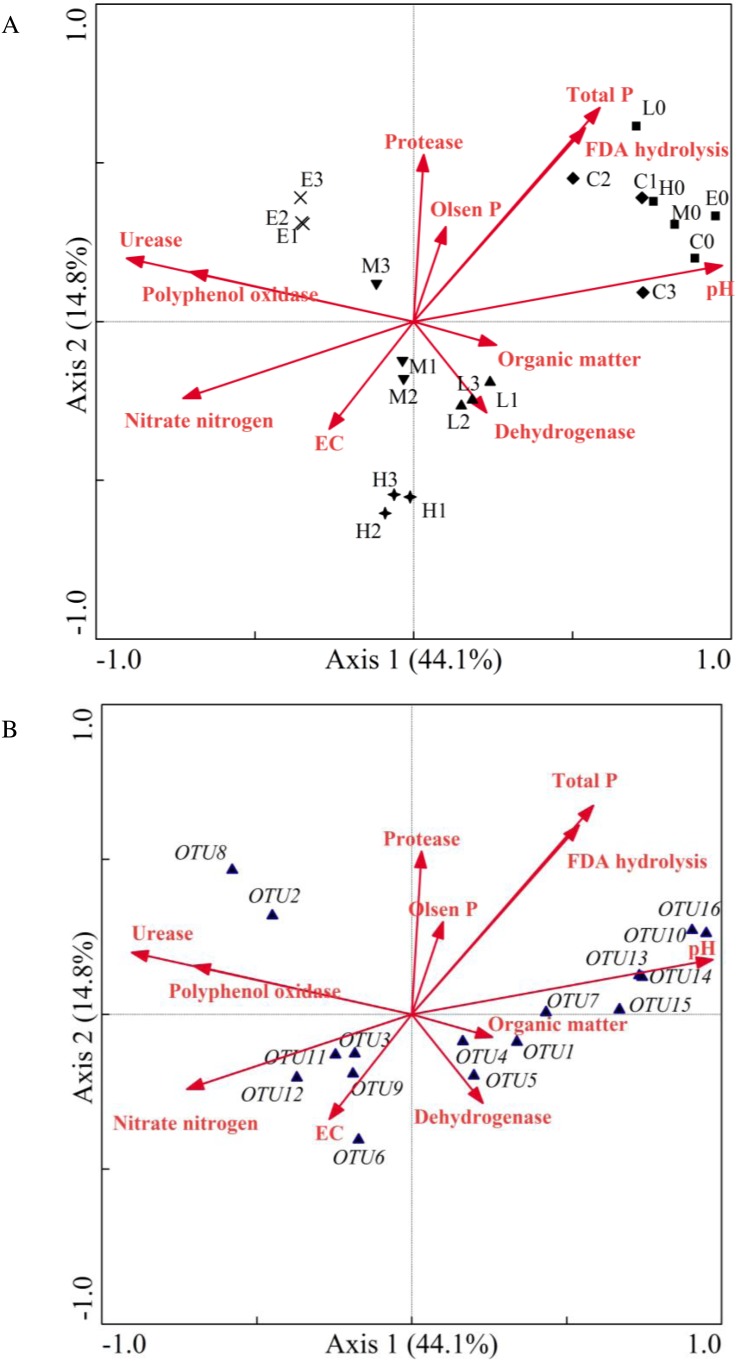
Canonical correlation analysis ordination plot indicating the relationship between the bacterial community and soil properties (A) or some frequent OTUs (B). (C, Control; L, 400 g m^−2^ day^−1^; M, 1,000 g m^−2^ day^−1^; H, 1,500 g m^−2^ day^−1^; E, 2,000 g m^−2^ day^−1^; 0 stands for the samples collected at T0; 1, 2 and 3 stand for the samples collected at T1, T2 and T3, respectively).

As shown in Fig. 6, samples of the initial group at T0 were gathered with samples of the C treatment at T2. The sample locations of the L, M, H and E treatments were significantly changed. The angle size between the arrows of environmental factors represented the correlation between environmental factors (Fig. 6A). The EC value, urease and polyphenol oxidase were significantly correlated with nitrate nitrogen. In addition, total phosphorus, FDA hydrolysis, Olsen phosphorus, and protease were grouped together, whereas pH, organic matter, and dehydrogenase had a greater distance. Moreover, the goodness of fit statistic for environmental variables indicated that ranking was highly correlated with pH, total phosphorus, urease, FDA hydrolysis, nitrate nitrogen, and polyphenol oxidase (*p* < 0.01) (see long arrow in Fig. 6A). The correlation between environmental factors and species information can be expressed by the angle between the environmental factor arrow line and the linking line which connected the species point and center point. Therefore, on the left part of Fig. 6B, the correlations between nitrate nitrogen, EC value and OTU3 (*Methylobacillus*), OTU9 (*Methylophilus*), OTU11 (*Methylophilus*), OTU12 (*Methylophilus*) were relatively large. Urease and polyphenol oxidase also had a closer relationship with OTU2 (*Methylophilus*) and OTU8 (*Methylovorus*).

## Discussion

### Soil characteristic impacts under CO_2_ leakage stress

The biological and biochemical processes in the soil are the important foundations of terrestrial ecosystems. Leakage of CO_2_ during the geological sequestration process could elevate the soil CO_2_ concentration (Österreicher Cunha et al., 2015; Šibanc et al., 2014). As a result, the soil physicochemical properties, soil enzyme activity and microbial community structure might be affected, thus influencing the terrestrial ecosystems (Awasthi et al., 2014). It is therefore essential to study the impact of CO_2_ leakage.

In this study, the tested soil was weak alkaline (pH = 8.15) at the beginning of the experiment. As the ventilation concentration increased, the soil pH value decreased to 7.29. Moreover, in the early part of the experiment, the pH of each treatment decreased rapidly and gradually tended to be gentle. These results implied a strong negative correlation between CO_2_ concentration and the pH value (*r* between −0.9 and −1). Sáenz de Miera et al. (2016) reported a similar result and concluded that the CO_2_ injection introduced a pH value decrease of the CO_2_ injection sites relative to a zero CO_2_-addition control. Fernández-Montiel, Pedescoll & Bécares (2016) showed that the soil pH changed from slightly acidic at the control site to acidic in higher CO_2_ flux sites. Furthermore, Chen et al. (2016) showed a similar negative concentration between the pH value and the CO_2_ flux. However, McFarland, Waldrop & Haw (2013) found that soil pH did not show a significant correlation with the CO_2_ flux. In contrast, ventilation also had an obvious effect on the EC value. The EC value of soil leaching solution can reflect the water-soluble salt content in soil. The injection of CO_2_ can affect the solubility of calcium carbonate, magnesium carbonate, and calcium sulfate, thus affecting the water-soluble salt content in soil leaching solution. The EC values of the C treatment with no CO_2_ flux were relatively stable throughout the experimental period, whereas, the EC value of CO_2_-addition treatments were affected by the injection time and concentration of CO_2_ gas. The overall trend showed that the greater the concentration of ventilation, the greater the EC values. As the increasing ventilation time, the water soluble CO_2_ in soil reached saturation, then the solubility of calcium carbonate, magnesium carbonate and calcium sulfate in soil might be no longer affected by the gas ventilation. Therefore, the EC values of experimental treatments began to decline after time T2. McFarland, Waldrop & Haw (2013) indicated that the soil EC dropped sharply with an increasing CO_2_ concentration. In China, Zhang et al. (2016) investigated the effect of CO_2_ leakage on the soil properties and found that soil pH decreased with an increasing CO_2_ leakage concentration, while the EC did not change.

Soil organic matter plays an important role in the terrestrial ecosystem, and it represents an important index to estimate the soil carbon storage and to evaluate the soil fertility and quality. In the current study, there was no significant correlation between organic matter and ventilation time or concentration. At first, the soil microorganisms could not adapt to the gas flux, so the organic matter content decreased sharply after aeration. However, the soil organic content increased from T1 to T4. This result indicates that microorganisms might adapt to the changing environment. However, when the gas flux was terminated, the soil organic matter content decreased sharply during the recovery period. It was speculated that the organic matter was degraded by surviving bacteria. In future research, we might apply ^13^C labeling to determine the carbon flow. Moreover, the organic matter content was positively correlated with the CO_2_ flux, which supports the findings of Sáenz de Miera et al. (2014) and Chen et al. (2016). These results also implied that injection of CO_2_ flux decreased soil pH value, and influenced the microbial activities, then might result in the change of soil organic matter content.

An increased CO_2_ concentration in soil can influence nitrogen content through affecting nitrogen fixation, mineralization, nitrification, denitrification, anaerobic ammonium oxidation, ammonia volatilization and other biochemical processes (Yue et al., 2007; Chen et al., 2014). On the whole, the nitrate nitrogen content increased with the increasing ventilation time and concentration from T0 to T4. A similar result has been represented by McFarland, Waldrop & Haw (2013). However, a contrary result was shown by Chen et al. (2016) that the nitrate nitrogen content decreased sharply along the elevated CO_2_ flux. In the non-planting soils, the organic matter might originate from microbial metabolic activities. The increasing CO_2_ concentration may lead to the decreasing quantity and activity of microorganisms, thus affecting the organic matter content. After the CO_2_ gas had been injected into the soil, an acidic and anaerobic environment might be formed. Then, the concentration of carbonate ions was potentially influenced and the change resulted in the concentration variation of ammonium ion (Blagodatskaya et al., 2010; Phillips et al., 2012). Moreover, the anaerobic environment may affect the anaerobic ammonium oxidation process. In the same way, the increase of soil CO_2_ concentration might promote nitrification, which could increase the nitrate nitrogen concentration in the soil (Ross et al., 2000; West et al., 2015).

Phosphorus is one of the indispensable nutrient elements for plant growth and development. It is easily fixed in the soil, while the utilization rate is low. Although the change of Olsen-phosphorus and total phosphorus did not show an obvious correlation with the CO_2_ concentration, their contents were lower under CO_2_-addition treatments than under control treatment. Phosphorus is a necessary element for microbial metabolism. Some soil microorganisms may produce acidic substances through metabolism, then dissolve some insoluble phosphates and apply them to their own metabolic processes. However, the phosphorus content in soil was related to soil organic matter content. As shown in Fig. 2C, the soil organic content increased with the increasing ventilation time from T1 to T4, whereas the change of Olsen-phosphorus and total phosphorus showed a decreasing trend (Figs. 2E and 2F). This result indicated that the soil phosphorus content might be negatively correlated with soil organic matter. It might be speculated that the CO_2_ ventilation influenced the soil organic matter and bacterial activity, thus influencing the change of total phosphorus and Olsen-phosphorus contents. Sáenz de Miera et al., (2014) reported that the total phosphorus was not well correlated with the CO_2_ flux, the value was greater in the high and extreme site when compared with the control site, but they did not discuss the result. Fernández-Montiel, Pedescoll & Bécares (2016) reported that the Olsen phosphorus significantly increased as the CO_2_ flux increased.

### Soil enzyme activity impacts of high CO_2_ concentration

Soil enzyme activities demonstrated different characteristics under CO_2_ stress. The FDA hydrolysis showed a significant decrease, which indicates that the increasing CO_2_ concentration restrained its activity. In addition, the inhibitory effects of CO_2_ concentration on the activities of protease and dehydrogenase were not significant. The dehydrogenase activity was related to microbial respiratory metabolism. It was then speculated that the complex change of dehydrogenase activity might be related to the inhibition of soil microbial respiratory metabolism under increasing CO_2_ stress. However, the increasing CO_2_ concentration showed different degrees of stimulation to promote the activities of polyphenol oxidase and urease. The activity of polyphenol oxidase increased considerably, while the increase of urease activity fluctuated. Sinsabaugh (2010) mentioned that the chemical oxidation during the experiment assay can cause higher polyphenol oxidase values. However, McFarland, Waldrop & Haw (2013) demonstrated that there was no significant change in polyphenol oxidase activity under an increasing CO_2_ flux. In another study, the researchers found that the elevated atmospheric CO_2_ concentration enhanced the protease activity, while polyphenol oxidase and urease activities remained relatively unchanged (Shi et al., 2016). Their study implied that elevated CO_2_ concentration improved the availability of soil organic content, and it increased the soil microbial activity. Then, it induced the changes of soil enzyme activities. It can be speculated that the elevated soil CO_2_ concentration decreased soil pH, organic matter content, total phosphorus and Olsen phosphorus content, resulting in the decrease of FDA hydrolysis activity. Moreover, it might be inferred that the increasing urease activity was associated with the elevated nitrate nitrogen content in the soil.

### Phylogenetic impacts of elevated soil CO_2_ concentration

Soil samples were collected for microbial analysis after one month of ventilation. The alpha and beta diversity analysis results showed that soil microbial community diversity decreased under CO_2_ stress. However, the abundances of adapted dominant species increased with the increasing ventilation time and soil CO_2_ concentration, while the microbial structure tended to be single. By means of species substitution, the soil microbial community might adapt to the regional environment caused by high CO_2_ concentration. It can be concluded that a local acid hypoxic soil environment might be formed under CO_2_ stress. This represents a great survival challenge to a variety of bacteria and could cause many aerobic bacteria to die, thus leading to the decrease of soil bacterial richness and diversity. Dunbar et al. (2014) examined the impact of 11 years of elevated CO_2_ on soil bacteria and contrasting results to the current study. They concluded that bacterial community in the 0–5 cm depth mineral soil demonstrated only minor changes after 11 years of elevated CO_2_.

As Fig. 4 shows, the distribution of phyla abundance differed under different CO_2_ stresses, implying a possible impact of soil CO_2_ flux. Results showed that dominant phylum of soil samples was *Proteobacteria*, which was promoted under the increasing soil CO_2_ concentration. Meanwhile, Šibanc et al. (2014) reported a contrasting result which stated that the abundance of *Proteobacteria* decreased with increasing CO_2_ concentration in spite of its dominant position. Sáenz de Miera et al. (2014) showed that a change in the abundance of the *Proteobacteria* phylum was less substantial under an increasing CO_2_ concentration at a naturally occurring CO_2_ gas vent. Moreover, it has rarely been mentioned in relevant literature that the abundances of genera *Methylophilus*, *Methylobacillus*, *Methylovorus* and *Methylotenera* belonging to the family *Methylophilaceae* (class *Betaproteobacteria*) sharply increased. Their growth substrates are generally with methyl groups. *Methylophilus* has been recognized as the aerobic methanol-oxidizing bacterium capable of degrading a variety of nitrogen-containing contaminants, such as methylamine, tetramethylammonium, and formamide (Kolb, 2009). Genera *Methylobacillus*, *Methylophilus* and *Methylovorus* have been assigned as the terrestrial obligate and restricted facultative *Methylobacteria*. The genus *Methylobacillus* was also presented to utilize methanol or methylamine as a sole source of carbon and energy (Gogleva et al., 2011). *Methylobacillus* sp. has been detected in riparian wetlands of Jiuduansha which could produce several oxidases that oxidize diverse compounds (Strom, Dinarieva & Netrusov, 2001; Hu et al., 2014). Moreover, Khan et al. (2015) proposed that the *Methylophilus* could be used as a candidate for explosive detoxification in a nitrogen-deficient environment. Furthermore, genera *Methylobacillus*, *Methylotenera* and *Methylovorus* have been identified as methanotrophs which was related with methane metabolism under elevated CO_2_ through the use of the whole genome metagenomic approach (Bhattacharyya et al., 2016). Fernández-Montiel, Pedescoll & Bécares (2016) also found the presence of *Methylocella palustris* in extreme flux which could produce CO_2_ from CH_4_ in an oxic environment. In contrast, *Variovorax* also showed a rising trend with an increasing CO_2_ concentration. Bablu & Sumathi (2015) reported that the whole cells of *Variovorax* sp. BS1 had the ability to biodegrade dimethyl phthalate (DMP). Above all, it might be speculated that increasing soil CO_2_ concentration might promote the increase of some methyl organic compounds in this experiment, which leads to a more suitable environment for these genera belonging to *Proteobacteria*. Furthermore, there is speculation that the injected CO_2_ was utilized to produce methane which finally was consumed by these *Methanotrophs*.

The *Acidobacteria* played a significant role in the soil ecological processes, and this diverse phylum was distributed widely in various natural environments (Tringe et al., 2005; Janssen, 2006; Liu et al., 2016). Based on 16S rDNA gene analysis, this phylum generally occupied 10–50% of the total soil bacterial communities (Fracchia et al., 2006; Penn et al., 2006; Lee & Cho, 2011). Liu et al. (2016) demonstrated that the abundances of subgroups Gp4, Gp6 and Gp7 were significantly positive in relation to the soil pH value. Our study showed a similar result that the abundance of *Acidobacteria* decreased with the decreasing soil pH which implied that the relative abundance of *Acidobacteria* was significantly and positively correlated with soil pH. Moreover, there was a decline in the subgroups Gp4, Gp6 and Gp7 of *Acidobacteria* abundances. The shift of *Acidobacteria* should be related to the changes in soil pH values due to the soil acidification after CO_2_ injection. Moreover, the phenomenon that the subgroups 4, 6, 7, 10, 11, 16, 17, 18, 22, and 25 were positively correlated with soil pH was presented in the broad-scale survey of *Acidobacteria* communities across 87 soils throughout North and South America by Jones et al. (2009). The occurrence of this phenomenon might be related to the different subgroups of *Acidobacteria*, or perhaps even the same subgroup might show different responses to the soil environmental factors. Sáenz de Miera et al. (2014) presented that the soil pH decreased and the abundance of *Acidobacteria* _Gp4 and *Acidobacteria* _Gp6 sharply declined as the CO_2_ flux increased. However, in another study Sáenz de Miera et al. (2016) showed that the relative abundance of *Acidobacteria* increased, and the abundances of classes Gp4 and Gp6 increased with a CO_2_ injection in Cubillos del Sil soil. On the contrary, the abundances of classes Gp4 and Gp6 did not change considerably under high CO_2_ injection in Hontomín soil. These phenomena indicated that it was impossible to determine whether the pH itself affected the shaping up of *Acidobacteria*, or whether the pH was indirectly related with the observed bacterial consortium changes through other environmental factors. Such environmental factors often co-vary together with the soil pH changes (Rousk et al., 2010). Previous studies have suggested that other soil parameters, such as soil C/N ratio, ammonium, phosphorus concentration, mineral element contents, and soil enzyme activity were also related with the *Acidobacteria* community composition (Naether et al., 2012; Navarrete et al., 2013; Zhang et al., 2014).

Sáenz de Miera et al. (2014) have reported that as the flux increased, the relative abundance of the *Chloroflexi* phylum increased while *Acidobacteria*, *Verrucomicrobia* and *Gemmatimonadetes* decreased. This result was slightly different from our result, which concluded that *Chloroflexi* phylum also decreased under CO_2_ stress. Usually, leakage of CO_2_ might result in an anaerobic environment, and the relative abundance of anaerobic bacteria such as *Bacteroidetes*, *Chloroflexi* and *Firmicutes* would show an increasing trend (Šibanc et al., 2014). However, *Bacteroidetes* did not show a significant relationship with the CO_2_ injection, whereas *Chloroflexi* and *Firmicutes* decreased with an increasing CO_2_ concentration. *Planctomycetacia* presented a notable increase after 14 days of CO_2_ exposure from a controlled CO_2_ sub-seabed leak in Ardmucknish Bay (Tait et al., 2015). In contrast, *Planctomycetes* phylum displayed a declining trend in our experiment.

Regarding the temporal variation shown in Fig. 5A, the PCoA results indicated that the bacterial communities changed throughout this experiment. Figure 5A also displayed that the C treatment at T0 was far away from the C treatment at Ti (*i* = 1, 2, 3). Whereas, in the Fig. 5B, there were just two major groups: one containing L and C samples and the other containing M, H, and E samples. As shown in Fig. 5A, the percentage explanation of PC1 (principal component) was 62.4%, indicating the relation between the samples on this axis. However, the percentage of PC2 explanation is just 8.1%, so the results obtained on this axis were less reliable in interpretation. Therefore, we used the Fig. 5B to verify that the L and C samples had higher similarity. Another explanation might be that climatic characteristics such as temperature and precipitation might also affect the bacterial communities. Seasonal effects have been observed by several authors for different experiments (Sáenz de Miera et al., 2014). This indicates that the separation between C1, C2 and C3 from C0 might be caused by climatic characteristics.

The CCA result indicated that the sample value of the CO_2_-addition treatments increased along the arrow direction of nitrate content, urease and polyphenol oxidase. However, the other environmental variables such as pH, total phosphorus, and FDA hydrolysis showed the opposite trend. In addition, the pH value with the longest arrow was most closely related to CO_2_ concentration, which suggested that this environmental factor was significant in inducing the differentiation of microbial community. All of these OTUs were closer to the L, M, H, and E groups than they were to the C group, which confirmed that these OTUs were significantly influenced by CO_2_ concentration. This also indicated the changes of environmental factors such as nitrate nitrogen, urease and polyphenol oxidase, which might bring about a more suitable environment for these OTUs. Moreover, the correlations between pH and OTU7 (*Lysobacter*), OTU10 (*Streptophyta*), OTU13 (*Thermosulfurimonas*), OTU14 (*Gp6*), OTU15 (*Subdivision3_genera_incertae_sedis*), OTU16 (*Kofleria*) were significant and the relative abundance of *Acidobacteria*_Gp6 was significantly and positively correlated with soil pH. Synthesizing Figs. 6A and 6B indicates that the arrows were longer for the OTU10 (*Streptophyta*) which belonged to *Cyanobacteria*, and the arrow was also longer for OTU16 (*Kofleria*) belonging to the phylum *Proteobacteria*. This implies that these two OTUs were important variables. Moreover, there was a high correlation between organic matter, dehydrogenase and OTU1 (*Sphingomonas*), OTU4 (*Massilia*), and OTU5 (*Janthinobacterium*). Unfortunately, all the OTUs had less correlation with protease, Olsen phosphorus, total phosphorus and FDA hydrolysis. The CCA results also indicated a gap between the control group and the groups under different CO_2_ gas stress conditions. All of these results confirmed that the observed changes of *Proteobacteria* and *Acidobacteria* phyla were significantly impacted by CO_2_ injection.

## Conclusion

Ventilation of CO_2_ gas significantly affected soil physiochemical properties and enzyme activity (lower pH and FDA hydrolysis activity, as well as increased nitrate nitrogen content and polyphenol oxidase activity), and resulted in lowered microbial diversity by favoring *Proteobacteria* phylum, such as *Methylophilus*, *Methylobacillus*, *Methylovorus*, and *Methylotenera*. The promotion of these organisms might be linked to the increasing some methyl organic compounds in this experiment under CO_2_ leakage stress. In contrast, the abundance of *Acidobacteria* decreased and the reason might be correlated with the comprehensive changes of soil parameters, such as soil phosphorus concentration, mineral element contents, and soil enzyme activity. Our data also indicated that the effect of CO_2_ leakage on the soil environment was significant, and that some of the effects were unrecoverable. Moreover, this study indicated that high CO_2_ leakage can impact the soil microbial community and pose a risk to the ecological heath of soil overlying CO_2_ storage reservoirs.

##  Supplemental Information

10.7717/peerj.4024/supp-1 Supplemental Information 1 Raw data for soil properties

10.7717/peerj.4024/supp-2 Supplemental Information 2 Phylum read

10.7717/peerj.4024/supp-3 Supplemental Information 3 Genus read

10.7717/peerj.4024/supp-4 Supplemental Information 4 Experimental figure

## Additional Information and Declarations

### Data Availability

The following information was supplied regarding data availability: The raw data has been supplied as a Supplementary File.
