# Supplementary figures and images for: Short-term effects of CO2 leakage on the soil bacterial community in a simulated gas leakage scenario
Source: PeerJ. 2017 Nov 14;5:e4024. doi: 10.7717/peerj.4024 (PMC5691795; doi:10.7717/peerj.4024)

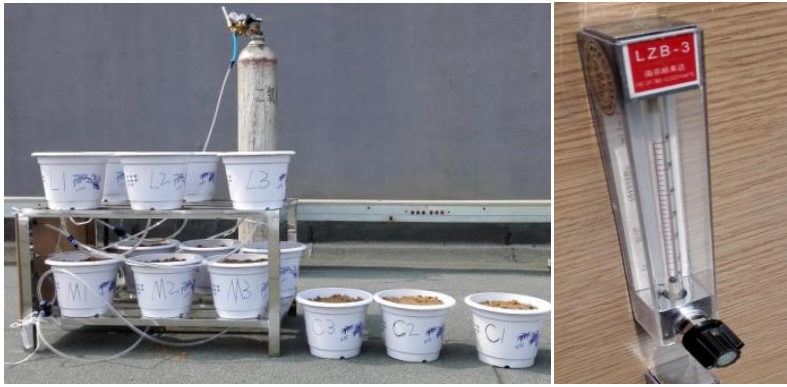

Profile of the experimental device

Supplement: Supplemental Information 4 [file peerj-05-4024-s004.pdf]
